# The efficacy of bedaquiline versus kanamycin in multi-drug resistant tuberculosis: A systematic scoping review

**DOI:** 10.4102/hsag.v26i0.1708

**Published:** 2021-11-29

**Authors:** Lisa Singh, Lehlohonolo J. Mathibe, Varsha Bangalee

**Affiliations:** 1 Discipline of Pharmaceutical Sciences, Faculty of Health Sciences, University of KwaZulu-Natal, Durban, South Africa; 2 Division of Pharmacology (Therapeutics), Faculty of Health Sciences, University of KwaZulu-Natal, Durban, South Africa

**Keywords:** DR-TB, bedaquiline, kanamycin, efficacy, treatment, outcomes, comparison, replacement

## Abstract

**Background:**

Drug-resistant tuberculosis (DR-TB) has become a serious cause of concern both on a global scale and in South Africa. It is associated with a lower successful treatment rate, thus creating a hurdle in achieving good treatment outcomes for patients.

**Aim:**

The aim of this study was to compare the efficacy of the drug kanamycin, an injectable aminoglycoside, to bedaquiline, a newer oral drug used to treat DR-TB.

**Methods:**

PubMed and Google Scholar, both of which are online databases, were extensively searched using the necessary keywords so that studies that were relevant to the scoping review were retrieved. A data-charting list was developed to extract the needed data for this scoping review.

**Results:**

The main findings of the scoping review showed that bedaquiline was highly efficacious in the treatment of DR-TB, and that it was a valuable addition in the treatment of DR-TB. The findings of the study also showed that kanamycin does not have good efficacy against DR-TB. and its use extends the treatment of DR˗TB.

**Conclusion:**

It stands to reason that bedaquiline replaces kanamycin in the DR-TB drug regimen as it was shown to be more efficacious and patients experienced better treatment outcomes in a shorter period of time. There were also fewer adverse effects associated with bedaquiline as compared to kanamycin.

**Contribution:**

Bedaquiline-based DR-TB therapy is more efficacious than aminoglycoside-based regimens which include kanamycin.

## Introduction

Tuberculosis (TB) is a preventable and curable disease caused by *Mycobacterium tuberculosis* (Alagna et al. 2020:1; Keshavjee & Farmer 2012:931). However, current tobacco smoking as well as poor patient adherence and compliance have resulted in emergence of drug-resistance tuberculosis (DR-TB) (CDC 2017; Wang et al. 2018:876). The DR-TB is a deadly disease caused by *Mycobacterium tuberculosis* strains which display *in vitro* resistance to two first-line TB therapy drugs: namely, isoniazid and rifampicin (Dookie et al. 2018:1138). As reported by the World Health Organization (WHO), globally the number of people that have multidrug˗resistant tuberculosis/rifampicin-resistant tuberculosis (MDR-TB/RR-TB) was 206 030 in 2019, which is an increase of 10% from 2018 (186 883). Globally, in 2019, about 3.3% of the new TB cases and 18% of TB cases that have been previously treated, had MDR-TB. In South Africa, in 2019, the estimated percentage of new cases with MDR-TB was 3.4% and the estimated percentage of previously treated cases with MDR-TB was 7.1% (WHO 2020). The resistant strain of TB has created a hurdle for the achievement of good treatment outcomes, as DR-TB is associated with lower treatment success and increased mortality (Zhao et al. 2019:1522–1523).

The current treatment of DR-TB is complicated, involving less efficacious and numerous drugs with multiple adverse effects (Ahmad et al. 2018:822). A time period of 9–20 months can be expected for the full treatment of DR-TB, and requires patients to take the anti-TB medication daily (WHO 2020) lengthy therapy may result in poor patient adherence and compliance (Lange et al. 2019:646).

For many years, aminoglycosides antibiotics, which inhibit bacterial protein synthesis, have been the main drugs of choice for the treatment of DR-TB (Serio et al. 2018:1). This group of antibiotics include drugs such as kanamycin, amikacin and capreomycin. As these drugs are injectables, patients are required to be hospitalised, in order for the drug to be administered. Aminoglycosides are associated with multiple detrimental adverse effects, such as irreversible hearing loss (Reuter et al. 2017:1114).

Therefore, after many decades, a new drug, bedaquiline, has been developed and introduced for the treatment of DR-TB. This medicine has shown good culture conversion during phase IIb clinical trials is associated with a good treatment response against DR-TB and is administered orally (Olaru et al. 2017:1). It is imperative that the efficacy of the injectable agents be compared to the efficacy of bedaquiline. This is because, the introduction of bedaquiline may result in an amended DR-TB treatment regimen, where it replaces the main injectable aminoglycosides, namely, kanamycin (Bastard et al. 2019:3). The aim of this study was to compare the efficacy of the drug kanamycin to bedaquiline. In order to fulfil this, the following questions were answered:

- In studies that revealed how efficacious bedaquiline is, how was this determined and presented?
- In studies that revealed how efficacious kanamycin is, how was this determined and presented?
- In studies that discussed bedaquiline and kanamycin, how were these drugs compared and what were the outcomes of the comparison?

## Methodology

### Scope of the search

A comprehensive search for articles was performed on 28 and 29 December 2020. An additional search was performed on 21 April 2021 to determine if any additional articles had been published. The search strategy was developed using a methodology paper that provided guidance for conducting a systematic scoping review (Peters et al. 2015:141–146). PubMed and Google Scholar databases were thoroughly searched by using keywords that were related to the efficacy of bedaquiline and kanamycin. In the search for studies that evaluated the efficacy of bedaquiline, studies that compared bedaquiline to the second line injectables (kanamycin) were identified. In the search for studies that evaluated the efficacy of kanamycin, the keyword DR-TB was included to provide relevant studies, pertinent to this scoping review.

### Inclusion and exclusion criteria

Studies were only included in the scoping review if the main focus of the studies were the efficacy of bedaquiline and/or kanamycin. Studies that compared bedaquiline to kanamycin or discussed the replacement of kanamycin with bedaquiline were included. Only studies published after 2012 were included as bedaquiline is a relatively new drug and studies that discussed the efficacy of bedaquiline were published mainly post 2012. To ensure that the analysis of results in studies that showed the efficacy of kanamycin were based on current standards, only studies published after 2012 were chosen for this scoping review. Studies that examined the efficacy of bedaquiline combined with another drug were excluded, as the efficacy of the drugs were discussed and not bedaquiline alone. It is to be noted that this does not apply to the background regimen of drugs that are given with bedaquiline. Additionally, studies that discussed kanamycin efficacy in other conditions beside DR-TB were excluded.

### Extraction and processing

A data charting list was developed and used to extract the relevant data for this scoping review. Data about the year of publication, source country/country of origin, aims/purpose of the study, study population and sample size, methodology, concept of the study, how outcomes were measured (if applicable) and key findings that relate to the review questions, were extracted.

### Results

**FIGURE 1:**
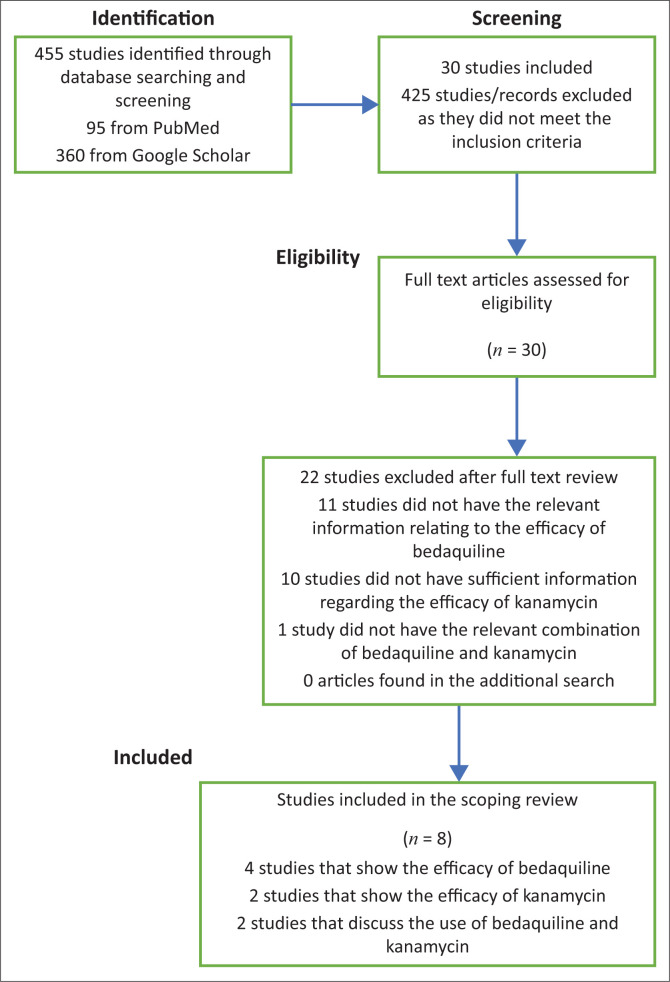
Summary of the searches and the processing of information.

Of the 455 results identified during the search, a total of eight relevant studies were selected. All of the studies were published after 2012 and met all the inclusion criteria. Figure 1 outlines the result of the search. No additional relevant studies were found in the additional search performed on 21 April 2021.

## Research topics

The following major research topics of the published studies were identified:

- The use and efficacy of bedaquiline in patients with DR-TB (Agnarson et al. 2020; Barvaliya et al. 2020; Guglielmetti et al. 2015; Sarin et al. 2019).
- The use and efficacy of kanamycin in patients with DR-TB (Cegielski et al. 2020; Van Altena et al. 2017).
- The implication of replacement of second line injectable anti-TB medication (kanamycin) with bedaquiline (Kashongwe et al. 2020; Zhao et al. 2019).

### The use and efficacy of bedaquiline in patients with drug-resistant tuberculosis

Four of the eight studies included in the scoping review reflect the efficacy of bedaquiline in DR-TB, along with the treatment outcomes for bedaquiline. The study by Guglielmetti et al. (2015) was conducted in Paris, France. The study conducted by Barvaliya et al. (2020) took place in Ahmedabad, India. Sarin et al. (2019) conducted their study at Delhi, India. The study conducted by Agnarson et al. (2020) was conducted in China. All of these were performed in patients who had confirmed DR-TB or pre-extensively drug resistant tuberculosis (pre-XDR TB). The Pre-XDR is defined as TB that is resistant to isoniazid, rifampicin and either fluroquinolones or a second-line injectable agent (Sarin et al. 2019:209–201). The researchers either conducted retrospective or prospective cohort studies, and the treatment outcomes of the patients who participated in these studies were recorded. As bedaquiline is a relatively new drug and its use is still monitored closely, most studies were performed to determine the early or initial outcomes with the use of bedaquiline.

All four studies indicated that bedaquiline resulted in a high rate of culture conversion from positive for DR-TB to negative. The culture conversion occurred in a relatively rapid period of time (in about six months). In all of the studies a very high percentage of patients had culture converted by six months to negative, indicating a favourable outcome of treatment with bedaquiline and that bedaquiline is effective in the treatment of DR-TB. Also, the results of these studies indicated that bedaquiline is associated with lower mortality rates. It is to be noted that bedaquiline was not administered as a standalone treatment in any of the studies; it formed part of an optimised background regimen of other re-purposed anti˗TB drugs. However, not all of these drugs were administered together as each of the studies had created their own optimised background regimen according to the WHO guidelines; although there were no great differences between the regimens. In the study conducted by Guglielmetti et al. (2015) patients received a background regimen combination of four of the following antibiotics: linezolid, para˗aminosalicylic acid (PAS), amikacin, imipenem combined with co˗amoxiclav, cycloserine, fluoroquinolone, ethambutol, pyrazinamide, ethionamide and clofazimine. In the study conducted by Barvaliya et al. (2020), patients received a combination of four of the following second line anti˗TB drugs: kanamycin/capreomycin, levofloxacin/moxifloxacin, ethionamide/cycloserine/PAS, linezolid/clofazimine/high dose isoniazid/clarithromycin, and pyrazinamide. In the study conducted by Sarin et al. (2019), most patients received high dose moxifloxacin (40%), second line injectable (73%), linezolid (90%), clofazimine (86%), ethionamide (58%) and cycloserine (60%). These studies suggest that a bedaquiline-based anti-TB medication regimen for the purpose of treating DR-TB has good culture conversion rate and are effective in achieving a good treatment outcome in patients who have DR-TB (Barvaliya et al. 2020:227–230; Guglielmetti et al. 2015:192–193; Sarin et al. 2019:211–212).

In a particular study that was conducted in China, a model was developed to determine the effect of bedaquiline on the treatment outcomes for DR-TB as well as the predicted incidence rate of DR˗TB from 2020 to 2040. The study model indicated that the use of bedaquiline decreased the incidence, prevalence and mortality that is associated with DR-TB, as compared to no use of bedaquiline which was associated with an increase in the incidence of DR-TB. Therefore, the study concluded that the use of bedaquiline is necessary to decrease the burden of DR-TB (Agnarson et al. 2020:6–7).

### The use and efficacy of kanamycin in patients with drug-resistant tuberculosis

The second line injectable agents for the treatment of DR-TB are the aminoglycosides, namely amikacin, kanamycin and capreomycin. Kanamycin is one of the aminoglycosides that has been used in the anti˗TB regimens and was developed in the 1950s (Cegielski et al. 2020:1). In the studies that were included in this scoping review (Cegielski et al. 2020; Van Altena et al. 2017), both used patient data to determine if kanamycin is effective in the treatment of MDR-TB. One study was a meta-analysis (Cegielski et al. 2020:2).

In the study by Cegielski et al., conducted in the United States of America, patients who were receiving each of the injectable drugs were compared to those who were receiving no injectable drugs (an oral treatment regimen). The injectable drugs that were included in this study were streptomycin, amikacin, kanamycin and capreomycin. In this study, kanamycin was most frequently used as compared to the other aminoglycosides. Kanamycin was not associated with any apparent benefit when compared to patients who did not receive any injectable drugs. Also, amikacin appeared to be more beneficial than to kanamycin and capreomycin. The authors concluded that kanamycin was associated with worse treatment outcomes than expected (Cegielski et al. 2020:2–7).

Although kanamycin is associated with nephrotoxicity and hearing loss, a study conducted by Altena et al. in the Netherlands showed that therapeutic drug monitoring can be used to help reduce the hearing loss associated with kanamycin (Van Altena et al. 2017:6–8). In this study, the treatment outcomes were good and only a low level (11.3%) of hearing loss occurred in the patients who were involved in this study. This may be because of the therapeutic drug monitoring (TDM)-guided dose that was given to the patients being lower than doses that were not TDM-guided. However, this dosage still resulted in an acceptable treatment outcome that did not present with severe hearing loss. Therefore, it is possible that kanamycin, when used with the TDM-dosage, allows for safer usage. Thus, kanamycin, when used with an adjusted dosage, can still exhibit efficacy in the treatment of DR˗TB with reduced adverse drug reactions.

**TABLE 1:** Summary of studies on the efficacy of bedaquiline, efficacy of kanamycin and the replacement of kanamycin with bedaquiline.

Reference	Year	Endpoint (measurement)	Major findings	Remarks
Guglielmetti et al.†	2015	Efficacy, safety, and tolerability of bedaquiline. No measurement specifications for outcome variables.	Regimens that contained bedaquiline resulted in a fast culture conversion rate within 6 months, suggesting the ability of bedaquiline-containing regimens to result in fast culture conversion in very difficult to treat patients.	Interim analysis of a French cohort involving 35 patients. Median age of the group was 39 years. Study performed in France.
Sarin et al.†	2019	Efficacy and adverse events. No measurement specifications for outcome variables.	Smear and culture conversion occurred early, indicating that bedaquiline-containing regimens are effective as they result in early conversion of both smear and culture tests.	Cohort of 290 patients that were drug resistant to tuberculosis. Patients had to be 18 years and older. Study performed in Delhi, India.
Barvaliya et al.†	2020	Treatment safety profile, adverse effects, efficacy of bedaquiline and optimised background regimen. Primary outcomes were specific and general adverse effects. Secondary outcomes were microbiological improvement and clinical improvement,	Good culture conversion rates. Found that bedaquiline-containing regimens well tolerated with slight extensively drug˗resistant tuberculosis prolongation. Lower mortality rates with good treatment outcomes.	Observational, continuous, prospective, single centre study in cohort of 127 patients who have DR-TB. Patients had to be more than 18 years of age. Study performed in India.
Agnarson et al.†	2020	Potential of bedaquiline-containing regimens to decrease the DR-TB burden, along with the incidence, mortality, and prevalence of MDR-TB. No measurement specification for outcome variables.	In baseline scenario, which excluded the use of bedaquiline, an increase in the DR-TB incidence, mortality, and prevalence. The scenarios that included bedaquiline, showed a decrease in incidence, mortality, and prevalence.	Study used a state-transition model. Data inputs were obtained from published literature, official TB statistics, WHO, World Bank, Population Division of the United Nations, WHO Global Health Observatory data repository. Study performed in China.
Altena et al.‡	2017	Pharmacokinetics/pharmacodynamics of the aminoglycosides (kanamycin/amikacin), adverse effects and clinical outcomes. No measurement specification for outcome variables.	Treatment outcomes in patients who received a lower, TDM-guided dose were acceptable. There was a lower percentage of hearing loss associated with the lower, TDM-guided dose.	Retrospective study that involved 80 patients. The median age of the patients was 30.5 years. Study performed in the Netherlands.
Cegielski et al.‡	2020	Each injectable drug compared with no injectable drug, XDR-TB, second-line injectable drugs compared with streptomycin, second-line injectable drugs compared with each other. No measurement specification for outcome variables.	In this study, amikacin was associated with better cure rates, mortality rate or both. Patients that received kanamycin had worse treatment outcomes than receiving no injectable drug.	Individual patient data meta-analysis of 12 030 drug-resistant TB patients. No measurement specification for outcome variables. Study performed in the United States of America.
Zhao et al.§	2019	Primary outcome measure being the proportion of patients that have an unfavourable outcome at the 12th month. Secondary outcomes were death, loss to follow-up, sputum culture conversion that was positive 6–12 months after treatment began was treated as treatment failure.	Substituting bedaquiline for second line injectables (aminoglycosides) resulted in better treatment outcomes. The substitution did not cause an increase in the mortality rates. There were differences in sustained culture conversion that supported the substitution of second line injectables. There were lower rates of culture reversion among the patients who were on bedaquiline,	Retrospective cohort study with 330 patients who had MDR-TB. The median age for patients who received bedaquiline was 42 and the median age for the patients who did not receive bedaquiline was 35 years. Study performed in South Africa.
Kashongwe et al.§	2020	Treatment outcomes defined according to the 2013 WHO reporting framework definitions except for failure and cure. Failure was any positive culture after 6 months during treatment, cure was defined by treatment completed without evidence of failure, and 3 or negative cultures taken at least 30 days apart during treatment.	Good culture conversion rate with bedaquiline. Patients presented with already established hearing loss which would have worsened if aminoglycosides were given. The shorter regimen with bedaquiline offers an alternative to the regimen that contains the second line injectables. Bedaquiline resulted in high therapy success.	Retrospective study that involved 39 patients. Patients had to be 18 years or older. Study performed in the Democratic Republic of the Congo.

**DR-TB, drug-resistant tuberculosis, MDR-TB, multidrug˗resistant tuberculosis; TB, tuberculosis; TDM, therapeutic drug monitoring; XDR-TB, extensively drug resistant tuberculosis; WHO, World Health Organization.**

† , denote studies that show the use of bedaquiline for patients with DR-TB and the efficacy of bedaquiline along with the treatment outcomes for bedaquiline;

‡ , denote studies that show the use of kanamycin in patients with DR-TB and the efficacy of kanamycin along with the treatment outcomes for patients who are treated with kanamycin;

§ , denote studies that show the replacement of second line injectable anti-TB medication with bedaquiline and the outcomes of this replacement.

### The implication of replacement of second line injectable anti-tuberculosis medication (kanamycin) with bedaquiline

Compared to bedaquiline, second-line injectables, are associated with lower successful treatment outcomes and a greater severity of adverse effects in the treatment of DR-TB. Therefore, the replacement of the second line injectables can only be done with a drug that will be effective and provide a high level of treatment outcomes, such as bedaquiline (Zhao et al. 2019:1522). In the studies that compared the aminoglycosides (kanamycin) and bedaquiline, patient data were used and the results from the patient’s treatment outcomes provided the study the data for comparison. One of the studies applied a retrospective cohort design (Zhao et al. 2019:1522–1523).

In the first study by Zhao et al., half of the study population received bedaquiline instead of a second line injectable (including kanamycin), while the other half received the standard treatment for DR-TB, which included the use of a second line injectable. The study concluded that the substitution of bedaquiline for kanamycin resulted in better treatment outcomes after 12 months of treatment when compared to the full course of DR-TB treatment regimen that contains second line injectables. Bedaquiline resulted in better culture conversion and reduced culture reversion supporting the use of bedaquiline in the DR-TB regimen.

The use of bedaquiline can allow for a shorter DR-TB regimen to be used without aminoglycosides. In a study conducted with patients who already had hearing loss in the Democratic Republic of the Congo, bedaquiline replaced kanamycin. This study was conducted by Kashongwe et al., and noted a better treatment outcome and good culture conversion (meaning samples taken from patients converted from positive for DR-TB to negative). It was found that the shorter regimen with bedaquiline offered a replacement or alternative to the longer regimen that involved the use of kanamycin (Kashongwe et al. 2020:4).

## Discussion

Bedaquiline is a relatively new anti-TB drug that is effective against DR-TB. It has a unique mechanism of action, and is administered orally, making it a much-needed option in the treatment of DR-TB (Gualano et al. 2016:43). Second-line injectable drugs, one of which is kanamycin, have been used in the treatment of DR-TB for a long time. This medicine is included in the treatment regimens for DR-TB but is associated with serious adverse drug reactions (Cegielski et al. 2020:1). Bedaquiline-containing regimens have shown fast culture conversions from positive for DR-TB to negative in 6 months. Most of the patients who have been placed on a bedaquiline-containing regimen, have shown good treatment outcomes and have not culture reverted to positive once testing negative. Also, bedaquiline is orally administered, therefore allowing for ease of administration for the patient. (Barvaliya et al. 2020:227–230; Guglielmetti et al. 2015:192–193; Sarin et al. 2019:211–212). The use of a bedaquiline-containing regimen can possibly allow for a shorter duration of the DR-TB treatment course, as shown by one of the included studies conducted in the Democratic Republic of the Congo (Kashongwe et al. 2020:4). It is thus evident from the studies presented in the scoping review relating to the efficacy of bedaquiline, that bedaquiline is highly efficacious against DR-TB and makes a valuable addition to the anti-TB arsenal of drugs. Bedaquiline also has adverse events that have to be monitored, such as corrected QT interval (QTc) prolongation, but this adverse event that can be monitored and corrected (Barvaliya et al. 2020:223).

Although kanamycin is also used in the treatment of DR-TB, it is not orally administered. It was noted that kanamycin did not exhibit substantial efficacy against DR˗TB. Many patients did not achieve good therapeutic outcomes with the administration of kanamycin. Instead, several patients experienced unfavourable outcomes because of the severe adverse effects that are associated with kanamycin, such as ototoxicity and nephrotoxicity (Cegielski et al. 2020:6–7). One of the studies, however, concluded that lower, therapeutic doses of kanamycin can achieve an acceptable treatment outcome with a reduced hearing loss in patients (Altena et al. 2017:6–8). This can possibly lead to the development of new regimen doses for the aminoglycosides that will be more efficacious and tolerable for patients.

When bedaquiline is directly compared to kanamycin, there is a clear superiority on the side of bedaquiline. When bedaquiline was used in the place of kanamycin, patients experienced better treatment outcomes in a shorter period of time, along with fewer adverse effects. Also, the treatment duration was reduced considerably when a bedaquiline-containing regimen was used in the place of kanamycin (Kashongwe et al. 2020:4; Zhao et al. 2019:1522–1523).

In view of the above, it can be concluded that bedaquiline is more efficacious and produces less adverse events than kanamycin. Furthermore, bedaquiline allows for better outcomes for the patient, along with ease of administration. As reported by Singh et al., bedaquiline has the possibility of improving the quality of life of patients with DR-TB (Singh, Kumar & Kushwaha 2021:25).

There are certain limitation to this study. As kanamycin is a drug that has been used for many decades, outdated articles had to be excluded that may have important results. This exclusion was performed so that studies that utilised current standards were included in this study. Also, there were no studies found where bedaquiline and kanamycin are directly compared without the use of any other drugs as a background regimen.

### Implication and recommendations

This scoping review has implications for both clinical practice and future research. From this study, it is evident that bedaquiline is more effective in treating DR-TB than kanamycin. Therefore, the replacement of kanamycin with bedaquiline will allow for better treatment outcomes for patients. However, research should be conducted on bedaquiline on its long-term use as it becomes integrated into the DR-TB regimen.

## Conclusion

Given the efficacy of bedaquiline against DR-TB, it stands to reason that bedaquiline should replace kanamycin. However, more studies should be performed to determine the efficacy of bedaquiline for DR-TB in multiple settings (high income to low income countries). As bedaquiline is a relatively new drug, also the efficacy and adverse effects of bedaquiline should be constantly monitored. Kanamycin, along with the other second line injectables, can possibly be repurposed by adjusting the doses in order to reduce the adverse effects that are associated with their use. Thus, bedaquiline is a valuable and life-saving inclusion in the treatment of DR-TB. This will reduce the burden of DR-TB on the healthcare system in the world.
